# Molecular Mechanisms of Differentiation of Murine Pro-Inflammatory γδ T Cell Subsets

**DOI:** 10.3389/fimmu.2013.00431

**Published:** 2013-12-05

**Authors:** Karine Serre, Bruno Silva-Santos

**Affiliations:** ^1^Faculdade de Medicina, Instituto de Medicina Molecular, Universidade de Lisboa, Lisbon, Portugal

**Keywords:** γδ T cells, T cell differentiation, interleukin-17, interferon-γ, transcription factors, cytokines

## Abstract

γδ T cells are unconventional innate-like lymphocytes that actively participate in protective immunity against tumors and infectious organisms including bacteria, viruses, and parasites. However, γδ T cells are also involved in the development of inflammatory and autoimmune diseases. γδ T cells are functionally characterized by very rapid production of pro-inflammatory cytokines, while also impacting on (slower but long-lasting) adaptive immune responses. This makes it crucial to understand the molecular mechanisms that regulate γδ T cell effector functions. Although they share many similarities with αβ T cells, our knowledge of the molecular pathways that control effector functions in γδ T cells still lags significantly behind. In this review, we focus on the segregation of interferon-γ versus interleukin-17 production in murine thymic-derived γδ T cell subsets defined by CD27 and CCR6 expression levels. We summarize the most recent studies that disclose the specific epigenetic and transcriptional mechanisms that govern the stability or plasticity of discrete pro-inflammatory γδ T cell subsets, whose manipulation may be valuable for regulating (auto)immune responses.

γδ T cells, which were discovered three decades ago (1–3), remain a very puzzling population of lymphocytes. Together with αβ T cells and B cells, they make up the three somatically rearranged lineages that are found in all jawed and also in jawless vertebrates (lampreys and hagfish) (4, 5), thus highlighting a strong evolutionary pressure to keep the three lymphocyte lineages together.

One of the most striking characteristics of γδ T cells is their inherent ability to very rapidly secrete pro-inflammatory cytokines. This is likely attributable to the functional maturity of discrete γδ T cell subsets, producing either IFN-γ or IL-17, that readily populate secondary lymphoid organs (as well as peripheral tissues) where they make a key contribution to “lymphoid stress surveillance” (6). We (7) and others (8, 9) have shown that these functional γδ T cell subsets develop in the murine thymus before migration to peripheral sites (10). This review outlines our current molecular understanding of the development and function of γδ T cell subsets that influence both innate and acquired immunity.

## Roles of IFN-γ and IL-17-Producing γδ T Cells in Immune Responses

By secreting large amounts of IFN-γ, γδ T cells participate in controlling infection through the activation of macrophages and cytotoxic lymphocytes. IFN-γ producing γδ T cells have been shown to play major protective roles during murine West Nile, herpes and influenza viral infections (11–13); *Listeria monocytogenes, Escherichia coli*, and *Bordetella pertussis* bacterial infections (14–18); and *Plasmodium chabaudi* and *Toxoplasma gondii* parasitic infections (19–22). Moreover, γδ tumor-infiltrating lymphocytes constitute a critical early source of IFN-γ that controls tumor development *in vivo* (23, 24).

With respect to the production of IL-17, γδ T cells are a key component of the defense against infections with *Mycobacterium tuberculosis, E. coli, L. monocytogenes, Staphylococcus aureus, Candida albicans*, and *Pneumococci* (18, 25–32). One of the main functions of these IL-17-producing γδ T cells is to enable extremely fast neutrophil recruitment at the site of infection.

On the other hand, IL-17-producing γδ T cells have pathogenic roles in various inflammatory and autoimmune disorders (and animal models thereof), including collagen-induced arthritis (CIA) (33), experimental autoimmune encephalomyelitis (EAE) (8, 34–38), chronic granulomatous disease (39), uveitis (40), ischemic brain inflammation (41), colitis (42, 43), and psoriasis (44, 45). Moreover, IL-17 also seems to promote angiogenesis and consequently tumor growth (46) and metastasis (47).

Therefore, from a therapeutic point of view, it is of utmost importance: (i) to define in detail the γδ T cell subset(s) that perform each given function; (ii) to understand the extracellular clues that regulate the development of each subset; and (iii) to identify the molecular program(s) of differentiation that control the acquisition and maintenance of a specific effector function.

Here we will essentially focus on mouse models, but to emphasize the relevance of studying specific murine effector γδ T cell subsets we will highlight their human counterparts. For a comprehensive review on the differentiation of human γδ T cells please refer to Ref. (48). Moreover, although the present review focuses on IFN-γ- and IL-17-secreting γδ T cells, we note that some γδ cell subsets produce other cytokines including IL-4, IL-5, IL-13 (49–51), IL-10 (52, 53), and IL-22 (54–56).

## Phenotypic Description of IFN-γ- or IL-17-Producing γδ T Cell Subsets

Functional γδ T cell subsets in the mouse have been traditionally defined by their TCR Vγ usage [please note that we use the nomenclature proposed by Heilig and Tonegawa (57)] and preferential tissue distribution. For example, epidermal Vγ5Vδ1 T cells are mainly associated with the production of IFN-γ (58), although they have also been shown to produce IL-17 in response to skin injury (59). Vγ6Vδ1 T cells that are present in the tongue, lungs, and reproductive tracts mainly produce IL-17. Moreover, Vγ1 T cells colonize the liver, spleen, and intestine preferentially secrete IFN-γ, whereas Vγ4 T cells, which recirculate through blood, spleen, and lymph nodes, and are also located in the lungs, favor IL-17 production. However, this dichotomy is not so strict as mouse Vγ4 T cells produce IFN-γ or IL-17 depending on the model studied (7, 60, 61).

**Figure 1:**
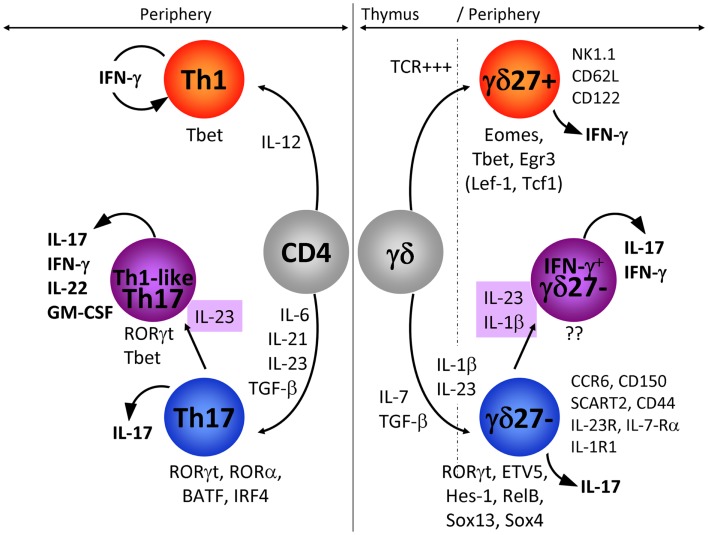
**IFN-γ-producing and IL-17-producing CD4 and γδ T cells**. In this figure, we have compared the extracellular signals and the transcriptional networks that regulate IFN-γ or IL-17 production in CD4 (left: Th1 and Th17) and γδ (right: γδ27^+^ and γδ27^−^) T cells. In addition, the expression pattern of markers specifically associated with IFN-γ-producing γδ27^+^ and IL-17-producing γδ27^−^ T cells is detailed. The emergence of IL-17^+^ IFN-γ^+^ T cells is highlighted for both CD4 and γδ T cells. Of note, the transcription factors in parenthesis (TCF1 and LEF1) below γδ27^+^ T cells have been proposed to inhibit IL-17 production in these cells.

Although a genome-wide transcriptional profiling of γδ thymocytes segregated the expression of some genes associated with IFN-γ or IL-17 production with selective Vγ chain usage (62), work from our laboratory (7), together with others (8, 63), has shown that γδ T cell functions are not mutually exclusive between Vγ1 and Vγ4 T cell subsets. Our collective efforts have identified CD27 and CCR6 as useful markers of discrete pro-inflammatory γδ T cell subsets: CD27 is expressed on IFN-γ-producing γδ T cells whereas IL-17-producing γδ T cells are CD27^(−)^ but express CCR6 (7, 54, 63) (see Figure 1 for further details). Of note, CD122 and NK1.1 constitute additional markers of IFN-γ-producing γδ T cells (8, 63). Consequently, we favor categorization of γδ T cell subsets based on their effector functions rather than on TCR Vγ usage (10). The definition of surface phenotypes associated with effector cell functions has greatly facilitated the dissection of the molecular mechanisms that control the differentiation of IFN-γ- or IL-17-producing γδ T cells.

## Differences in Cytokine Production between γδ and CD4 T Cells

One of the main differences between cytokine production by γδ and CD4 T cells resides in the spontaneous release of cytokine by γδ T cells, which strikingly contrasts with the delayed response of naïve CD4 T cells. This can be explained by γδ T cells exiting the thymus already functionally competent to produce either IFN-γ or IL-17 (7–9, 64), whereas CD4 T cells require a long differentiation program in peripheral lymphoid organs that consists of activation, intense proliferation, and induction of transcription factors that selectively control the profile of cytokines produced (65). As CD4 T helper cells have been extensively studied, it is reasonable to question if the programs of differentiation that prevail in CD4 T cells also operate in γδ T cells. Here we will focus on the molecular mechanisms that govern the differentiation of naïve CD4 T cells into IFN-γ-producing (Th1) and IL-17-producing (Th17) cells, as counterparts to CD27^+^ (γδ27^+^) and CD27^−^CCR6^+^ (γδ27^−^) γδ T cell subsets, respectively.

## Environmental Cues that Govern the Acquisition of Types 1 or 17 Effector Functions

Upon peripheral activation, naïve CD4 T cells are polarized toward the Th1 fate in the presence of IL-12 (66). As yet, there is no precise information as to the role of IL-12 in the development of γδ27^+^ T cells although IL-12 (in synergy with IL-18) induces the production of IFN-γ by γδ27^+^ T cells expressing NK1.1 (63). Our unpublished data suggest that IL-15 and, to a lesser extent IL-2, strongly promote IFN-γ production by γδ27^+^ T cells (Barros-Martins et al., manuscript in preparation).

Th17 polarization entails TGF-β, IL-6, and IL-1β, whereas IL-23 is required for maintenance and expansion (67–69). Although still controversial, the development of IL-17-producing γδ T cells in the thymus (and their maintenance in the periphery) appears to be dependent on TGF-β but mostly independent of IL-6 (9, 70–73). Unexpectedly, IL-7 induced rapid and substantial expansion of IL-17-producing γδ27^−^T cells (74). Furthermore, they require IL-23 and IL-1β for peripheral expansion and local induction of IL-17 (30, 75, 76). This is clearly evidenced by the significant reduction in IL-17-secreting γδ T cell numbers following *L. monocytogenes* infection in IL-23^−/−^ and IL-23R^−/−^ mice (72, 77) or in IL-1R1^−/−^ mice upon EAE induction (36). It was also shown that IL-18 synergizes with IL-23 to promote IL-17 production by γδ T cells (78). IL-17 production by γδ T cells can be triggered independently of TCR signaling (36, 54, 76), but it is worth noting that a small subset of CD44^+^CD62L^+^ γδ T cells (a phenotype associated with γδ27^+^ cells; see Figure 1) selectively recognized phycoerythrin via the TCR and became CD44^+++^CD62L^−^ cells that produced IL-17 (79). In this system too, propagation of the IL-17-response by PE-specific γδ T cells relies on IL-23. Finally, it has been shown that IL-17 derived from CD4 T cells is a negative regulator of IL-17^+^ γδ T cell development in adult thymus (64), underlying the potential danger that large numbers of these pro-inflammatory cells likely represent to the host.

## Transcriptional Regulation of Cytokine Production in γδ and CD4 T Cells

During Th1 polarization of naïve CD4 T cells, IL-12 activates STAT4 (80), but it is unclear if this IL-12/STAT4 axis plays any role in IFN-γ production by γδ27^+^ T cells. The “master” transcription factor that regulates the production of IFN-γ in CD4 T cells is T-bet (81, 82). Whereas Th1 differentiation is fully abrogated in the absence of T-bet, γδ27^+^ T cells only partially require T-bet to produce IFN-γ (83–85). Other transcription factors that have been proposed to play major roles in γδ T cells include Eomes and Egr3 (58, 84), although the potential cooperation between these three transcription factors within specific γδ T cell subsets still needs to be clarified.

Th17 differentiation relies on cytokines that target STAT3 and lead to the expression of the master transcription factor retinoic-related orphan receptor γt (RORγt) (86) that synergizes with RORα (87), together with IRF4 (88) and BATF (89) to propagate IL-17 production. *In vivo* Th17 cell differentiation also involves the aryl hydrocarbon receptor (AhR) (90, 91). All together this led to the concept that a specific transcriptional network is operating during initiation and stabilization of the Th17 phenotype (92).

IL-17 production by γδ27^−^T cells is also strictly dependent on RORγt (70, 85, 86, 93). However, the similarities between the Type 17 program of γδ and CD4 T cells end with this transcription factor, since STAT3 and IRF4 have been shown to be dispensable for the differentiation of IL-17^+^ γδ T cells (93, 94). Of note, detection of IL-17^+^ γδ T cells in STAT3-deficient mice further suggests that IL-6, IL-21, and IL-23 are unlikely to play major roles for their development, although they may be involved in peripheral reactivation of these γδ cells. AhR has also been shown to be dispensable for IL-17 but required for IL-22 production by γδ T cells (54). Finally, our unpublished data show that IL-17-producing γδ T cells are generated in the absence of RORα or BATF (Barros-Martins et al., manuscript in preparation). Thus, many transcription factors that are essential for Th17 development are not required for the differentiation of their IL-17^+^ γδ T cell counterparts.

In fact, γδ27^−^T cells appear to rely on distinct molecular pathways to regulate their production of IL-17. Namely, several transcription factors such as Sox13 and Sox4 (95, 96), Hes-1 (93), RelB (97), ETV5 (98) along with the kinase Blk (99), selectively participate in IL-17 production by γδ T cells. On the other hand, TCF1 and LEF1 are negative regulators of IL-17 expression in γδ T cells (96).

These data clearly highlight that distinct mechanisms govern the production of IFN-γ and IL-17 in CD4 and γδ T cells (Figure 1). Further studies are warranted to precisely delineate the molecular components of the Types 1 and 17 programs of γδ T cells.

## Stability Versus Plasticity of γδ T Cell Subsets

Initially studies suggested that the segregation between IL-17 and IFN-γ production that emerged in the thymus appeared to be stable in the two γδ T cell subsets, including in peripheral lymphoid organs and upon challenge with infectious agents *in vivo* (7, 76). Furthermore, incubating the γδ27^+^ cells in Th17 conditioning milieu, or the γδ27^−^ cells in Th1 conditioning milieu, failed to “convert” their cytokine production profile (63, 85). It was therefore assumed that, due to thymic “functional pre-commitment,” murine γδ T cells harbored little plasticity, in stark contrast with CD4 T cells (100).

To get further insight into the molecular mechanisms of stable commitment of the γδ27^+^ and γδ27^−^ T cell subsets to their respective effector functions, we undertook the first genome-wide comparison of the chromatin landscape of these two γδ T cell subsets. We analyzed the distribution of methylation marks on histone H3 (H3). Methylation of lysine 4 (H3K4me2/3) signs actively transcribed loci, whereas methylation of lysine 27 (H3K27me3) represses the accessibility for the transcriptional machinery (101, 102). As expected, we found that gene loci associated with IL-17 production harbored active histone modifications only in γδ27^−^T cells. By contrast, and to our surprise, gene loci associated with IFN-γ showed active H3K4me2 profiles in both γδ T cell subsets. Furthermore, whereas *Il17* and related genes were exclusively transcribed in γδ27^−^cells, *Ifng* and genes that control its expression were transcribed in both γδ27^+^ and γδ27^−^T cells (although to a lesser extent in the latter subset). Thus, *Ifng* and “Type 1” factors are epigenetically and transcriptionally primed for expression in both γδ27^+^ and γδ27^−^T cells, which led us to hypothesize that γδ27^−^ T cells could acquire IFN-γ expression under specific conditions.

## Identification of γδ IL-17^+^ IFN-γ^+^ Double Producers

By performing a series of *in vitro* experiments, we found that IL-1β strongly synergizes with IL-23 to induce IFN-γ expression specifically in IL-17-producing γδ27^−^cells (Figure 1). Importantly, epigenetic and transcriptional polarization of IL-1R1 and IL-23R predicted the responsiveness of γδ27^−^ cells, but not γδ27^+^ cells, to these two inflammatory cytokines.

This plastic behavior of γδ27^−^T cells was also observed *in vivo*, as IL-17^+^ IFN-γ^+^ γδ27^−^cells could be found in the peritoneal cavity of mice bearing ovarian tumors (85). Moreover, these cells have been detected in the brain of mice suffering from early stages of EAE (103); and in the mesenteric lymph nodes of mice infected with *L. monocytogenes* (104).

Double producing IL-17^+^ IFN-γ^+^ γδ T cells have also been characterized in humans. Thus, while a fraction of neonatal and adult Vγ9Vδ2 T cells incubated with IL-6, IL-1β, and TGF-β in the presence of TCR agonists produced IL-17A, the addition of IL-23 resulted in IFN-γ co-production (105). Moreover, IL-17^+^ IFN-γ^+^ cells of both Vδ1 and Vδ2 subtypes were found in the circulation of HIV^+^ patients (106).

Thus, although their precise physiological relevance is still to be established, IL-17^+^ IFN-γ^+^ double producers can clearly be a distinct component of the γδ T cell response in scenarios of infection, cancer, and autoimmunity.

## CD4 IL-17^+^ IFN-γ^+^ Double Producers and Their Biological Relevance

IL-17^+^ IFN-γ^+^ double producers have been well characterized in the CD4 T cell compartment (Figure 1). In particular, both murine (107, 108) and human (109–111) Th17 cells often show plasticity in acquiring IFN-γ production. Strikingly, these IFN-γ^+^ (Th1-like) Th17 cells have been strongly associated with pathogenicity in murine (107, 112, 113) and human (114) autoimmune syndromes. The molecular determinants of pathogenicity of Th1-like Th17 cells are still controversial, with studies either implicating T-bet and IFN-γ (108, 112, 115) or not (116–118). Nonetheless, it is clear that IL-23 is a major driver of Th1-like Th17 cell pathogenicity (108, 112, 117).

Similar studies on *in vivo* models should now explore the potential pathogenic role of γδ IL-17^+^ IFN-γ^+^ double producers. This notwithstanding, it has been proposed that, in response to *L. monocytogenes*, IL-17^+^/IFN-γ^+^ producing γδ27^−^ cells become memory cells capable of providing enhanced protection against recall infection (104). Thus, γδ IL-17^+^ IFN-γ^+^ double producers may potentially play host-protective versus pathogenic roles in distinct disease models, which will be an interesting topic for future research.

## Concluding Remarks

As a population, γδ T cells perform a wide variety of functions, but discrete subsets have more restricted effector properties. Although thymic development endows a significant fraction of murine γδ T cells with a “pre-determined” effector function, recent data provide strong evidence for functional plasticity in the periphery (particularly for γδ27^−^T cells).

Several fundamental questions remain unanswered. Is functional plasticity restricted to γδ T cells located in secondary lymphoid organs or does it extend to subsets that populate epithelial tissues/mucosas? Why did γδ T cells and CD4 T cells evolve different transcriptional networks to regulate the production of the same pro-inflammatory cytokines? What are the specific roles of γδ IL-17^+^ IFN-γ^+^ double producers in models of infection, cancer, and autoimmunity? More globally, it will be important to dissect the physiological stimuli that drive the activation of effector γδ T cells. It is particularly puzzling that we still know so little about the role of the TCRγδ, and the identity of its ligands, in the differentiation and activation of functional γδ T cell subsets. Answering these questions will improve our understanding of γδ T cell physiology and likely provide new avenues for the design of immunotherapeutic approaches.

## Conflict of Interest Statement

The authors declare that the research was conducted in the absence of any commercial or financial relationships that could be construed as a potential conflict of interest.
